# Motherhood in childhood: addressing reproductive health hazards among adolescent married women in India

**DOI:** 10.1186/s12978-016-0171-7

**Published:** 2016-05-04

**Authors:** Shraboni Patra

**Affiliations:** International Institute for Population Sciences, Govandi Station Road, Deonar, Mumbai, 400088 Maharashtra India

**Keywords:** Adolescent pregnancy, Stillbirth, Abortion, Pregnancy complications, Delivery complications, DLHS, India

## Abstract

**Background:**

In India, due to the high prevalence of child marriage, most adolescent pregnancies occur within marriage. Pregnancy and childbirth complications are among the leading causes of death in girls aged 15 to 19 years. Hence, adolescent pregnancy is a serious health threat to young women in India.

**Methods:**

The study focuses on the level and trends of adolescent pregnancy rate (per thousand currently married adolescent women) in India in the last two decades, based on cross-sectional data from three different periods, DLHS-1 (1998–99), DLHS-2 (2002–04) and DLHS-3 (2007–08). Further, the determinants of adolescent pregnancy and its effects are analyzed using the DLHS-3 data, which used a multi-stage stratified systematic sampling design. The sample size of this study was 18,709 pregnancies that occurred to 14,006 currently married adolescent (15–19 years) women. Chi-square tests and logistic regression were used to examine the association between pregnancy outcomes (live birth vs. abortion/stillbirth) and health complications with socioeconomic variables and maternal-child health (MCH) service utilization.

**Results:**

During the periods of 1998–99, 2002–04 and 2007–08, the rate of adolescent pregnancy was 427, 467 and 438 respectively. In 2007–08, the proportion of live births (vs. stillbirth or abortion) was significantly higher among older adolescents aged 18–19 years (OR = 1.25, 95 % CI (1.08–1.44), *p* < 0.001) than among younger adolescent women of 15–17 years. The proportion of live births was also higher among women having 10 years or more education (OR = 1.26, 95 % CI (1.01–1.56), *p* < 0.01). The prevalence of live birth was significantly higher among women who had received some delivery advices (OR = 1.38, 95 % CI (0.96–1.95), *p* < 0.01), had consumed iron/folic acid tablets, (OR = 1.37, 95 % CI (0.89–2.11), *p* < 0.05), had received Tetanus Toxoid injection (OR = 2.29, 95 % CI (1.25–4.19), *p* < 0.001), while those with assisted vaginal delivery were significantly less likely to have a live birth (OR = 0.38,95 % CI (0.21–0.68), *p* < 0.001). Adolescent women had 66.6 % delivery complications (i.e. any one problem) vs. 62.5 % among adult women (20–24 years), (*p* < 0.001).

**Conclusion:**

Stillbirth and abortion are more prevalent among younger adolescents than among older adolescents, and among all adolescents than among adult women. Delaying the first birth until age 20 appears to benefit both mothers and babies. Access to reproductive health services; timely and quality family planning services and safe abortion and delivery advice; tetanus toxoid and iron/folic acid for those married adolescents who do become pregnant could improve health outcomes.

## Background

Adolescent pregnancy or ‘motherhood in childhood’, is one of the most serious health threats to young women in India. About 16 million women 15–19 years give birth each year, which accounts for approximately 11 % of all births globally [1]. The average adolescent birth rate in the middle- income countries is two times higher and in the low-income countries is five times higher as compared to high-income countries [1]. Adolescent pregnancy can lead to morbidities (such as sexually transmitted diseases), mental disorders (such as depression) as well as higher neonatal mortality [2]. These are the high-risk births for both mother and child. They are also high-cost births when the associated negative effects on quality of life and the role of women in society are considered.

The term ‘adolescence’ is variously defined. It is “the period of transition from childhood to adulthood, encompassing both the development to sexual maturity and to psychological and relative economic independence” [3]. According to the World Health Organization (WHO), the transition from childhood to adulthood may be referred to as ‘adolescence’ or ‘teenage’, which is known as the period of 10–19 years [4].

Unlike many other countries, having a child outside marriage is uncommon in India. Adolescent fertility in India occurs mainly within the context of marriage. As a result of early marriage, about half of all young women are sexually active by the time they are 18 years old; and almost one in five by the time they are 15 years [5]. As compared to women who married as adults, women who married as minors are more likely to report early, frequent and unplanned pregnancies (typically as the consequence of not using contraceptives), which have been consistently linked to increased risk of maternal and infant morbidity and neonatal mortality [6, 7]. Their age-related risk is compounded by malnutrition and inadequate antenatal care (ANC) [8]. Several medical complications, such as preterm birth, poor maternal weight gain, pregnancy-induced hypertension, anemia, and sexually transmitted diseases are associated with adolescent pregnancy [2, 9].

In India, child marriage was outlawed (Child Marriage Restraint Act) in 1929, and it set the minimum age of marriage for women as 15 years. The legal age for marriage was increased to 18 years for girls in the amended law in 1978. However, the marriage of adolescent girls at below their legal age is still prevalent in India [10]. While, abortion has been legally accepted in India in 1972, limited availability and poor quality of services have kept safe abortion beyond the reach of poor women. Of the estimated five million induced abortions that occur annually in India, only half a million are performed under the health services network [11]. Induced abortion is somewhat more likely to occur among adolescents than among older women [5]. Most of the adolescent girls are not aware of family planning methods, and even if they are aware, they do not have easy access to family planning services or fail to utilize them. Seeking an abortion is neither approved by family members nor socially sanctioned.

Adolescent pregnancy or early childbearing and unsafe abortion is a multifaceted problem in India affecting families, health care professionals, educators, government officials, and youth. In India, pregnancy among adolescents after a marriage has social approval but negative maternal and neonatal outcomes. Therefore, the level and trend of adolescent pregnancy in India with respect to present sociocultural settings needs in-depth study and substantial discussions. In addition, there are factors such as inadequate prenatal care, illiteracy, and poor socioeconomic conditions which affect the outcome of pregnancy among adolescent women. Lack of prenatal care and delivery care can contribute to higher risks of neonatal morbidity and mortality [2]. Another key determinant is contraceptive use, which is important for a better reproductive health in the adolescent years when pregnancy and childbirth should be avoided as long as possible. All these factors and their effects on pregnancy among adolescents need an intricate debate in the Indian context and require the utmost attention of researchers and policymakers.

The present study focuses on the level and trend of adolescent pregnancy in India. Further, the important determinants (such as sociocultural factors and health care services utilization) of adolescent pregnancy, the impact of early pregnancy on adolescent health in terms of complications during pregnancy, and problems during delivery are also investigated.

## Methods

### Data and sample

This is a secondary analysis of datasets from three rounds of District Level Household and Facility Survey (DLHS). The Ministry of Health and Family Welfare (MoHFW), Government of India (GOI) has designated the International Institute for Population Sciences (IIPS), Mumbai as the nodal agency for conducting the DLHS. It is a household survey at the district level.

Three different time periods data, District Level Household and Facility Survey-I (DLHS-1, 1998–99), DLHS-2 (2002–04) and DLHS-3 (2007–08) were used to show the level and trend of adolescent pregnancy in India. Further, the DLHS-3 (2007–08) data is used to show the determinants and consequences of adolescent pregnancy in India. The DLHS-3 data used a multi-stage stratified systematic sampling design. The details of the sampling procedure are available in the DLHS reports [12–14].

In the present study, women aged between 15 and 19 years are considered as adolescents, and pregnancy occurred to them is termed as ‘adolescent pregnancy’. A total of 18,709 pregnancies, that occurred to 14,006 currently married adolescent women (15–19 years) is used for analysis. The sample of adolescents of 15–17 and 18–19 years of age are 2701 and 11,305 respectively. Further, a sample of 73,455 currently married adult women of 20–24 years is taken to carry out a comparative analysis of pregnancy and delivery complications. In DLHS-I and DLHS-II, only information on the pregnancy of currently married women is available. Hence, pregnancy among unmarried adolescents is not addressed in the present study.

### Variables

Several simple and compound variables have been constructed for analyzing the data. The variables used in the present study have been divided into two categories:

#### Predictor variables

The present study has used several predictor variables, which are re-coded for the purpose of analyses, and effective comparison of the results. They are: age group of women (15 to 19 in single year group, further sub-divided as younger adolescents 15–17 years and older adolescents 18–19 years); place of residence (rural, urban); age at consummation in years (less than 18 years, and 18 and above years); marital duration in years (less than 2 years and 2 years and above); education level of women in completed years of schooling (uneducated or < 5 years, 5–9 years, and ≥ 10 years; husband’s education level in completed years (uneducated or < 5 years, 5–9 years, and ≥ 10 years); contraceptive prevalence rate (CPR) for any method (yes or no); work status of women (yes or no); wealth quintile (lowest, second, middle, fourth and highest); Religion (Hindu, Muslim and Others (includes Christian, Sikh, Buddhist, Jain, Other, None); Caste (Scheduled Caste (SC) and Scheduled Tribe (ST), Other Backward Caste (OBC) and Others include Brahmin and others); number of antenatal care (ANC) visits (< 3 visits, ≥ 3 and above); ANC received in the first trimester (yes or no), received advice to go to health facility for pregnancy for last pregnancy (yes or no); received delivery advice for the last pregnancy (yes or no); Consumed iron and folic acid (IFA) tablets or syrup at least for 90 days (yes or no); received Tetanus Toxoid (TT) injection (yes or no); type of delivery (normal, caesarean, or by an instrument or assisted).

#### Dependent variables

Dependent variables used in the study are also re-coded purposively. Pregnancy outcome is categorized as two parts, live birth and stillbirth/abortions, where abortion includes both spontaneous and induced. To see the effect of heath care services utilization on adolescent pregnancy, the pregnancy outcome variable is again re-coded into live-birth and still-birth, but excluding abortion. The other dependent variables are: any pregnancy complications (which includes any common problems, i.e. swelling of hands, feet and face, paleness, giddiness/weakness, excessive vomiting, hypertension/high blood pressure or any major problems or any problems with the fetus; any major pregnancy complications (visual disturbance, excessive fatigue, convulsion, not from fever, jaundice, excessive bleeding and abnormal vaginal discharge); problems with fetus (weak or no movement of the fetus and abnormal position of the fetus); complications during delivery (including premature labour, excessive bleeding, prolonged labour, obstructed labour, breech presentation, convulsion/high blood pressure and others).

### Statistical analysis

Bivariate and multivariate analyses are used to see an association between predictor variables and the outcome variables.

The level and trends of adolescent pregnancy in India are shown based on the three different time periods (DLHS-1, DLHS-2, and DLHS-3). The analyses were conducted only for currently married adolescent women, considering their last pregnancy. The pregnancy rate among adolescents is shown in a number of pregnancies per thousand adolescent women. The author performed further analyses in two stages. In the first stage, differentials in pregnancy outcomes among currently married adolescent women are observed according to their demographic and socioeconomic characteristics, such as age group of women, age at consummation, marital duration, place of residence, education level of women and their husbands, work status, caste, religion, wealth quintile of women. In the second stage, effects of women’s age (i.e. 15–19 years and 20–24 years) on complications during pregnancy and delivery are assessed.

The logistic regression model was used to analyze the effect of selected socio-economic factors on pregnancy outcome among adolescent women. Binary logistic regression was used to estimate the adjusted effect of background characteristics on pregnancy outcome. Chi-square tests were performed to determine the level of significance of the correlation between women’s age and the pregnancy and delivery complications. Statistical Package for the Social Sciences (SPSS), version 20 (IBM Corp, Armonk, New York) was used for data analysis.

## Results

### Levels and trend of adolescent pregnancy during the last two decades in India

Table 1 shows the trend of adolescent pregnancy rates since 1998 in India. It was found that the rate of total pregnancy among currently married adolescent women increased in India from 427 (per thousand women) in 1998–99 to 467(per thousand women) in 2002–04, followed by a decrease in 2007–08 to 438 (per thousand women).

**Table 1:** Adolescent pregnancy in India

Period	^a^Pregnancy per thousand currently married women aged 15–19 years	Women (*n*)
DLHS- I (1998–99)	427	14,255
DLHS-II (2002–04)	467	39,892
DLHS-III (2007–08)	438	34,346

^a^Only last pregnancy considered

### Factors determining pregnancy outcomes among currently married adolescent women in India

Table 2 summarizes the results of bivariate and multivariate analyses showing pregnancy outcome of currently married adolescent women by their background characteristics. Here, live birth is considered as a successful pregnancy outcome, whereas stillbirth and abortion (spontaneous or induced) are considered as unsuccessful pregnancy outcomes or pregnancy loss. In India, the percentage of successful pregnancy outcome among adolescent women was 88.9 %, whereas 11.1 % pregnancies resulted in stillbirth (2 %) and abortion (9.1 %). The proportion of stillbirth and abortion was significantly higher among younger adolescents than among older adolescents (13.1 % vs.10.6 %, OR = 1.25, 95 % CI (1.08–1.44), *p *< 0.001), women who had never used any contraception (12.2 % vs.6.2 %, OR = 0.47, 95 % CI (0.40–0.56), *p *< 0.001) and women with ≥ 10 years of education (11.5 % vs. 9.8 %, OR = 1.26, 95 % CI,(1.01–1.56), *p *< 0.01). Further, the prevalence of stillbirth and abortion was higher among adolescents from higher economic status (15.1 % vs. 7.6 % OR = 0.04, 95 % CI (0.32–0.53), *p *< 0.001) and Hindu religion (11.4 % vs. 7.7 % OR = 1.91, 95 % CI, (1.41–2.59), *p *< 0.001).

**Table 2:** Pregnancy outcomes among currently married women of 15–19 years age group by their background characteristics, India, 2007–08

Background characteristics	Pregnancy outcome				Total pregnancy (*n*)	OR (95 % CIs for Expβ)
	Live birth	Pregnancy loss^a^				
		Total	Stillbirth	Abortion^b^		
Women’s age (years)						
15	83.7	16.3	1.9	14.4	285	–
16	85.6	14.4	1.9	12.5	889	–
17	87.8	12.2	1.6	10.6	2329	–
18	88.7	11.3	2.3	9.0	6094	–
19	89.8	10.2	1.9	8.3	9112	–
15 to 17	86.9	13.1	1.7	11.4	3503	1.0
18 to 19	89.4	10.6	2.0	8.6	15,206	1.246*** (1.081–1.437)
Residence						
Rural	89.4	10.6	2.1	8.5	16,601	1.0
Urban	86.5	13.5	1.8	11.7	2108	0.932 (0.794–1.093)
Age at consummation (years)						
Below 18	89.5	10.5	2.0	8.5	16,501	1.0
18 & above	85.1	14.9	1.7	13.2	2208	0.770*** (0.653–0.906)
Marital duration (years)						
Less than 2	86.9	13.1	1.8	11.3	8142	1.0
2 & above	90.5	9.5	2.2	7.3	10,567	1.164** (1.027–1.319)
CPR for any method						
Yes	93.8	6.2	0.8	5.4	3331	1.0
No	87.8	12.2	2.4	9.8	15,378	0.473*** (0.398–0.562)
Women’s education level (in completed years)						
Uneducated or less than 5	90.2	9.8	2.2	7.6	10,617	1.0
5 to 9	87.0	13.0	1.9	11.1	6517	0.893* (0.788–1.012)
10 & above	88.5	11.5	1.4	10.1	1575	1.257** (1.011–1.562)
Husband’s education level (in completed years)						
Uneducated or less than 5	89.5	10.5	2.0	8.5	12,514	1.0
5–9	88.9	11.1	2.4	8.7	1944	0.879 (0.732–1.055)
10 & above	87.4	12.6	1.7	10.9	4251	1.050 (0.913–1.206)
Women’s work status ^e^						
Working	89.8	10.2	2.0	8.2	1972	1.0
Not working	88.8	11.2	2.1	9.1	11,844	1.037(0.882–1.218)
Wealth quintile ^e^						
Poorest	92.4	7.6	2.1	5.5	4439	1.0
Second	89.7	10.3	2.3	8.0	5120	0.726*** (0.607–0.868)
Middle	88.2	11.8	2.2	9.6	4457	0.613*** (0.511–0.736)
Fourth	86.5	13.5	1.5	12.0	3406	0.531*** (0.436–0.647)
Richest	84.9	15.1	1.6	13.5	1279	0.414*** (0.323–0.531)
Religion						
Hindu	88.6	11.4	1.9	9.5	15,058	1.0
Muslim	89.4	10.6	2.6	8.0	2676	1.142 (0.973–1.340)
Others	92.3	7.7	1.3	6.4	975	1.911*** (1.410–2.590)
Caste						
SCs/STs^c^	89.7	10.3	1.9	8.4	7455	1.0
OBCs^d^	88.8	11.2	2.1	9.1	7727	1.037 (0.910–1.181)
Others	87.6	12.4	1.9	10.5	3527	0.944 (0.801–1.111)
India	88.9	11.1	2.0	9.1	18,709	

^a^Unsuccessful outcome of pregnancy, ^b^Abortion includes both spontaneous and induced, ^c^The Scheduled Castes (SCs) and Scheduled Tribes (STs) are the official designations given to various groups of historically marginalized people, recognized in the Constitution of India. During the period of British rule, they were known as the Depressed Classes. In modern literature, the SCs are sometimes referred to as ‘Dalits’, whereas STs are mentioned as ‘Adivasis’ (i.e. traditional forest dwellers). The SCs and STs comprise about 16.6 % and 8.6 % respectively of India ’s population (Census,2011), ^d^OBC represents other backward classes, ^e^Total excludes missing cases; ***p< 0.001; **p< 0.01; *p< 0.05.

### Status of maternal and child health (MCH) services received by adolescent married women

Table 3 represents the effects of ANC and delivery care on pregnancy outcome of adolescent women. Results of bivariate logistic regression show that prevalence of successful pregnancy outcome (live birth) was significantly higher among all married adolescents who had received delivery advice (OR = 1.38, 95 % CI (0.98–1.95) *p *< 0.01), consumed iron and folic acid (IFA) at least for 90 days (OR = 1.37, (95 % CI (0.89–2.11), *p *< 0.05) and received TT injection (OR = 2.29, 95 % CI (1.25–4.19), *p *< 0.001) as compared to the reference population. Women who experienced delivery by instrument or by medical assistance (OR = 0.38, CI (0.21–0.68), *p *< 0.001), were less likely to give live birth.

**Table 3:** Percentage of currently married women of 15–19 years, experienced live/still birth by received Antenatal Care (ANC) and delivery care services for last birth, India 2007–08

ANC and delivery care services	Pregnancy outcome		Total women (*n*)^#^	OR (95 % CIs of Expβ)
	Live birth	Stillbirth		
Times of ANC received				
Less than 3	98.0	2.0	7487	1.0
3 and above	98.7	1.3	6519	1.118 (0.801–1.560)
ANC received in 1^st^ trimester				
No	98.1	1.9	8333	1.0
Yes	98.6	1.4	5673	1.159 (0.843–1.595)
Received advice to go health facility for pregnancy				
No	98.2	1.8	5728	1.0
Yes	98.7	1.3	4857	1.154 (0.830–1.606)
Received delivery advice^†^				
No	98.1	1.9	5675	1.0
Yes	98.8	1.2	4911	1.381** (0.975–1.954)
Consumed IFA^a^ at least for 90 days				
No	98.1	1.9	11,500	1.0
Yes	99.0	1.0	2506	1.369* (0.888–2.112)
TT^b^ injection received				
No	97.7	2.3	3737	1.0
Yes	98.5	1.5	10,269	2.291*** (1.253–4.187)
Delivery types/methods^†^				
Normal	98.4	1.6	12,713	1.0
Caesarean	98.5	1.5	879	1.037 (0.541–1.987)
By instrument or assisted	95.9	4.1	410	0.377*** (0.211–0.675)
India	98.3	1.7	14,006	

^#^Un-weighted frequency; ^†^Total excludes missing cases; ***p< 0.001, **p< 0.01, *p< 0.05, ^a^Iron and Folic Acid, ^b^Tetanus Toxoid

### Early pregnancy and reproductive health risk among adolescent married women

Table 4 shows that pregnancy complications and delivery complications were high among all adolescent women as compared to adult women aged 20–24 years. During pregnancy, adolescent women experienced more complications (62.4 %) than the adult women (59.9 %, *p *< 0.001). About 48 % of adolescent women had any major problems related to pregnancy as compared to (45.3 %, *p *< 0.001) adult women. Moreover, fetuses of adolescent women had more problems (9.7 %) than of adult women (9.2 %, *p *< 0.05). The study shows adolescents also had more problems during delivery. About 67 % adolescents experienced at least one problem during delivery, significantly more than the adult women did (63.1 %, *p *< 0.001).

**Table 4:** Percentage of currently married women of 15–24 years, experienced complications during their last pregnancy and delivery by age, India, 2007–08

Age (years)	Women had complications during pregnancy						Women had complications during delivery		Women (*n*)
	Any problem^a^ (%)	*χ* ^2^ test	Any major problem^b^ (%)	*χ* ^2^ test	Problem with foetus^c^ (%)	*χ* ^2^ test	Any problem^d^ (%)	*χ* ^2^ test	
15 to 17	63.9		48.8		9.5		67.0		2701
18 to 19	62.0		47.8		9.7		66.5		11,305
Total	62.4	3.853**	48.0	1.407	9.7	0.084	66.6	0.181	14,006
15 to19	62.4		48.0		9.7		66.6		14,006
20 to 24	59.5		44.8		9.1		62.5		73,455
Total	59.9	42.302***	45.3	46.302***	9.2	3.663*	63.1	74.614***	87,461

***p< 0.001, **p< 0.01, *p< 0.05, ^a^Women experienced any one of the common problems (swelling of hands, feet and face, paleness, giddiness/weakness, excessive vomiting, hypertension/high blood pressure), major problems and other problem during pregnancy, ^b^Women experienced any major problem (visual disturbance, excessive fatigue, convulsion not from fever, jaundice, excessive bleeding and abnormal vaginal discharge) during pregnancy, ^c^Women experienced problems during pregnancy related to either weak or no movement of foetus or abnormal position of the foetus, ^d^Women experienced any one of the following problems during delivery: premature labour, excessive bleeding, prolonged labour, obstructed labour, breech presentation, convulsion/high blood pressure and others

## Discussions

In India during the last two decades, the rate of adolescent pregnancy has slightly decreased, followed by a rapid increase. The present study found that the proportion of stillbirths and abortions were significantly higher among younger adolescents as compared to older adolescents (18–19). The pregnancy loss or undesirable and adverse pregnancy outcome (i.e. stillbirth and abortion) was significantly higher among all adolescent women from the highest wealth quintile than among those belonging to the lowest wealth quintiles, and the odds of having a stillbirth/abortion increased significantly with each step in wealth quintile.

The level of education of the respondents also showed a statistical difference with the prevalence of stillbirths and abortion, where stillbirth and abortion are found to be significantly higher among women having secondary education, but statistically lower for those with 5–9 years of education as compared to < 5 years. Although still birth is directly associated with poor maternal health and more prevalent among women from socio-economically disadvantaged background, the prevalence shows little difference from the prevalence among rich and highly educated adolescent women. The reason could be, women with previous abortion experience have elevated risks for stillborn and prematurity [15]. However, induced abortion may predispose to negative future pregnancy outcomes, including a higher rate of spontaneous abortion and ectopic pregnancy [16] among wealthier and higher educated women. Further, sex-selective abortion of female foetuses is more common in the educated and wealthier households in India. Presumably, the reason is that they can afford an ultrasound and abortion services more readily than the uneducated and poor women [17]. It is also evident that the probability of reporting of a miscarriage increases with the level of education of women [18]. In India, safe induced abortion is subjected to the level of knowledge of women, availability, affordability and perspective of providers of quality abortion services at the authorized health centres, located mainly in the urban areas [19]. Young women are particularly vulnerable to poor sexual and reproductive health in general, and they have especially poor access to safe abortion services, which leads to delays in obtaining services and reliance on unsafe providers [20, 21]. Besides, desire to defer first child or acceptance of family planning or spacing being more prevalent among women with higher income and education level [20].

In India, wide variation in the reproductive health status of adolescent women shows that state or district level strategies are obligatory to provide services to adolescent women for reproductive health needs. The use of contraception among married adolescents in India is despondently low among all groups. A study has found that the extent of adolescent fertility has been declined from 100 per 1000 in 1971 to 52 per thousand in 1999 in India. According to the World Bank, the adolescent fertility rate in India has been declining and is found 84, 82, 79 and 77 per 1000 women in 2008, 2009, 2010 and 2011 respectively [22]. The present study has shown that the rate of adolescent pregnancy is much higher as compared to the rate of adolescent fertility in India, which is a clear indication of a considerable number of pregnancies that are unsuccessful (i.e. abortion and stillbirth). Since 1971, the proportion of adolescent fertility to total fertility rate has been decreased from 9.7 % to 8.1 %. Besides, the prevalence of abortion and stillbirths among adolescent women has been increased in India in the past three decades and the rate considerably varies from state to state [23].

Further, stillbirth and unsafe abortions have an adverse effect on women’s health, particularly in their teenage. During the adolescent period, women are physically immature and psychologically unprepared to experience the long lasting blemish of an unsuccessful pregnancy on their physical and mental health. Again, the amalgamation of poor nutrition and early childbearing exposes young women to serious health risks during pregnancy and childbirth [24]. Complications during pregnancy and childbirth are the leading causes of mortality of girls aged 15–19 years in developing countries [25]. This study found slight differences, though statistically significant, in the prevalence of major pregnancy complications and problems during delivery between women of age group 20–24 and 15–19 years. It is also observed that women who became pregnant at the age below 20 years, experienced more prenatal complications compared to adult women. This finding is supported by the literature where it is reported that children born to adolescent mothers face a higher risk of dying than those born to women aged 20–24 years [10]. Further, the high rate of prevalence of any one of common complications experienced by adolescents during pregnancy (62.4 %) and delivery (66.6 %) are subjected to their broad definition, and these complications can be fatal and may affect maternal and neonatal outcome [26, 27]. It is also found that pregnancy complications and delivery complications are much more prevalent among teenage women than among older women [28]. The present study also found low prevalence of live birth and high prevalence of stillbirth among adolescents who had undergone an assisted delivery. There is also possibility of induced labour or assistance required to deliver a still-born baby. Hence, preventing pregnancies to women younger than 20 may improve neonatal outcomes.

A study has found that preterm delivery and perinatal complications most likely contribute to the risk of perinatal death in the poor and disadvantaged populations in developing countries, especially for the deliveries occurring outside hospitals or health care facilities [29]. Here it is worthy to mention that abortion-related causes of maternal mortality account for 8 % of all maternal deaths in India [30]. Hence, pregnancy prevention strategies and improvement of healthcare interventions may be crucial to reduce adverse pregnancy outcomes among adolescent women in India [31].

In India, undesirable outcomes of pregnancy among adolescents can be attributed not only to low maternal age but to their relatively disadvantaged socioeconomic background. In families living in poverty and other disadvantaged segments, the decline in the rate of adolescent pregnancies will not eliminate negative social and health effects. It requires a potentially productive strategy for widening the pathways out of poverty or, at the very least, not exacerbates the hindrance imposed by social disadvantages [32].

Hence, there is a need for an immediate endeavor to decrease the incidence of adolescent pregnancy and to minimize the risk of pregnancy and birth to those adolescent women who do become pregnant. Efforts need to be directed towards strict enforcement of laws to eliminate marriage of adolescents in India and delay first childbirth. For those who do not or cannot delay the first birth, the findings of our study and others [10, 28] indicate that access to quality health services and ANC visits where women can receive at least one TT injection, adequate IFA consumed for at least 90 days or more), and delivery advice significantly improve live birth outcomes. Additional care for pregnant adolescents during ANC should be taken to make sure that a minimum number of antenatal visits with high-quality content are made. Appropriate and adequate counseling on different antenatal services, delivery options, and risks, and family planning should be discussed during ANC.

### Limitations

Although, DLHS-3 is the last available district level, nationwide, cross-sectional data in India, it limits author’s ability to establish severe and adverse effects of early pregnancies on maternal and child health. The long-term health consequences of adolescent pregnancy on mother and child, could not be incorporated into the study due to the unavailability of data. Since the data is cross-sectional in nature, it does not allow for proper ascribing of causality and associations between explanatory variables and pregnancy outcome [33]. The broad definitions of “complications during pregnancy”, “complications during delivery” and “negative/adverse pregnancy outcome” in the data may not provide actual prevalence. Further, self-reported diseases prevalence suffers from recall bias and is subjected to women’s level of knowledge and willingness to report [34]. Hence, the negative outcome of pregnancy may be over or underestimated. Since present analyses are based on women’s report of complications rather than clinically confirmed complications, these definitions may have low validity for estimates of the prevalence of clinically relevant conditions [35].

## Conclusion

The prevalence of stillbirth and abortion is much higher among adolescent married women as compared to adult women in India. The study shows that prevalence of stillbirth is higher among adolescent women who have not received proper ANC advice and among those who have not experienced delivery done by any assistance or by an instrument. Hence, proper monitoring of ANC services and the assistance received for delivery by adolescent pregnant women is urgently needed to improve maternal health and to reduce neonatal mortality rate in India.

The results indicate that program and strategies at the national level need to be implemented to protect the reproductive rights of adolescent women, especially those from the disadvantaged background, and to screen their health problems (due to early pregnancy) in India. The provision of sex education in schools might contribute to increased knowledge of contraception, increase the ability to negotiate for contraceptive use, and, perhaps, contraceptive effectiveness among adolescents.

Both infant and maternal mortality rates might be significantly reduced if married adolescent women could be better educated. Further, adolescent women could be encouraged to adopt small family norm by postponing their first birth and by following proper birth spacing. Hence, avoiding childbearing during the adolescent period might allow young women to complete their education and help them to take the advantage of work opportunities. Moreover, postponement of the first pregnancy among married adolescents in India may aid to long-term health benefits to them, and may contribute to the country’s economic growth by reducing health expenditure on maternal and child morbidity.

### Ethical statement

The study has used anonymous public use data sets with no identifiable information on the survey participants. The data considered all the ethical issues, and informed consent was obtained from all the respondents while collecting the information. Therefore, no ethics statement is required for this work.
